# Materialism, the Dark Triad Traits, and Money Management among Undergraduate Students

**DOI:** 10.11621/pir.2024.0204

**Published:** 2024-06-01

**Authors:** Dmitriy S. Kornienko, Milena V. Baleva, Nadezhda P. Yachmeneva

**Affiliations:** a *Russian Presidential Academy of National Economy and Public Administration, Moscow, Russia*; b *Perm State University, Russia*

**Keywords:** materialism, the Dark Triad (DT), money management, personality, narcissism, Machiavellianism, psychopathy, Material Values Scale (MVS)

## Abstract

**Background.:**

Previous studies have assumed that a materialistic value orientation is correlates with personality traits such as honesty, neuroticism, and agreeableness. Less is known about the relationship between features of a materialistic orientation such as acquisition centrality, acquisition as the pursuit of happiness, and possession-defined success, and the Dark Triad traits. This article presents a study on the relationship between materialism, the Dark Triad traits (Machiavellianism, narcissism, and psychopathy), and money management.

**Objective.:**

The study aimed to investigate whether groups exhibiting various combinations of materialism and Dark Triad traits have disparities in financial control and accountability, which serve as indicators of money management.

**Design.:**

Questionnaire-based surveys were conducted online, with a total of 442 undergraduate students age 18 to 25 participating. The participants filled out the Short Dark Triad measure, the Material Values Scale, and the Money Management Scale, in addition to providing their demographics.

**Results.:**

Four combinations of materialistic and Dark Triad traits were revealed (Bright and Dark Materialists and Non-materialists). Bright and Dark Materialists were more self-centered and manipulative than Non-materialists. Strong materialism, paired with the lowest degree of Dark Triad characteristics, resulted in the worst financial management. On the contrary, individuals with low materialistic tendencies in addition to high Dark Triad traits tended to have better ability to managing their finances.

**Conclusion.:**

It is possible to assume that materialism is a strategy for obtaining riches, positions, and reputation at the cost of others in the case of “dark” personalities. Nevertheless, those with low levels of materialism and low Dark Triad characteristics showed better abilities to handle their finances in terms of control and responsibility.

## Introduction

Materialism is defined by Richins and Dawson (Richins & Dawson, 1992) as “the importance a person places on possessions and their acquisition as a necessary or desirable form of conduct to reach desired end states” (p. 307). According to the value-oriented approach to materialism (Richins, 2004; Richins & Dawson, 1992), this concept contains three elements or values: acquisition centrality, acquisition as the pursuit of happiness, and possession-defined success. Materialistic individuals believe that the quantity and quality of their material goods may measure their personal success. Possessing and consuming things can be their primary life goal and crucial to their life satisfaction and well-being.

A comprehensive review of materialism studies showed positive associations between materialistic values and compulsive consumption behavior (Kasser, 2016) or emotional buying (Donnelly et al., 2013). Materialistic individuals strived to have more and better things than others in order to gain positive self-appraisal and affect (Martin et al., 2019).

Concerning social life, high materialism is positively associated with shorter and low-quality relationships, low empathy, and manipulativeness (Kasser, 2016; Ouyang et al., 2020). In their relationships, materialists are oriented to external factors (appearances and status), and are self-centered (less giving, less engaged with relationships) (Tatzel, 2002). The link between materialism and well-being has been explained by self-determination theory (Ryan & Deci, 2000). People who prefer materialistic values show poor satisfaction levels in relation to psychological demands for autonomy, competence, and relatedness, which leads to low levels of well-being (Christopher et al., 2009; Dittmar et al., 2014; Kasser, 2016).

### Materialism and personality traits

Materialism has shown negative associations with traits in the HEXACO model, particularly with Honesty-Humility, but also with Agreeableness, Openness to Experience, and Conscientiousness (Ashton & Lee, 2008). The Big Five traits also correlate variously with materialistic values (*e.g.,* extraversion correlates positively) (Ashton & Lee, 2008). The combination of high extraversion and neuroticism with low openness and agreeableness predicts materialism (Otero-López & Villardefrancos, 2013). A recent study of materialism and personality traits revealed the mediating role of neuroticism and narcissism in connection with materialism and well-being (Górnik-Durose & Pyszkowska, 2020).

### Materialism-personality types

Different types of materialism, depending on their combination with HEXACO traits, were extracted by M. Górnik-Durose and I. Pilch (2016). These researchers identified two types of materialistic individuals, “Peacocks” and “Mice,” who differ primarily in their levels of extraversion and emotionality (neuroticism). The “Mice” types are more concerned with money as a source of stability and fulfillment of their desires and aspirations. “Peacocks” are a more narcissistic type who use money and luxury items to promote themselves. The time perspective of these two types of materialistic personalities differs as well. Materialists of the “Peacock” type have a present-hedonistic time perspective, whereas “Mice” materialists have a past-negative time perspective (Watson, 2020).

These studies support the idea of a dual-nature model of materialistic personality proposed by M. Górnik-Durose and I. Pilch (2016). The dual nature of materialism is rooted in two contrasting life experiences: 1) avoiding scarcity and 2) seeking to show off (Górnik-Durose & Pilch, 2016); and it is exposed in personality traits and the individual’s evaluation of past, present, or future experience, and well-being (Watson, 2020). The concept of materialist types intersects with the “money worlds” hypothesis (Tatzel, 2002) in money spending, allowing us to identify “Mice” as thrifty spenders and “Peacocks” as free spenders.

### Materialism and the Dark Triad

The desire for money, status, and prestige are the significant motivating factors for people with a configuration of narcissism, Machiavellianism, and psychopathy, the combination known as the Dark Triad (Paulhus & Williams, 2002). Each of these traits may indicate a different approach to acquiring material goods and possessions. Machiavellianism is associated with maximizing long-term personal benefits, and is correlated with representing money as an indicator of success, wealth, and motivational factors (Maggalatta & Adhariani, 2020). Psychopathy is manifested as reckless impulsivity in gaining advantages along with taking needless risks for minimal gain. Narcissism expresses itself as over-self-confidence and is associated with drives for reward-seeking and novelty (Jones, 2013).

Highlighting the common core of Honesty-humility and the Dark Triad traits (Machiavellianism, narcissism, and psychopathy), K. Lee (Lee et al., 2013) analyzed the effectiveness of HEXACO and the Big Five traits, in combination with the Dark Triad, in predicting the money factor in personality make-up (materialism and conspicuous consumption). The Dark Triad traits added more predictability than the Big Five traits (Lee et al., 2013).

Other research has revealed that Dark Triad traits and its facets accounted for 36% of the variance in materialism and 21–32% of the variance in materialism facets (Pilch & Górnik-Durose, 2016). Individuals high in narcissism and Machiavellianism demonstrate a materialistic orientation, but materialism cannot be the motivational drive for psychopaths in general. However, the combination of boldness (as a psychopathic feature) and narcissism increases the materialistic orientation. In evaluating the incremental validity of the Dark Triad over the HEXACO traits in measuring materialism, the same study found that adding narcissism, Machiavellianism, and psychopathy enhanced the predictive value of Honesty-Humility when Agreeableness and Extraversion were removed from the model. Despite that, the DT characteristics accounted for just a 3% percentage of variance in addition to the personality dimensions mentioned above (Pilch & Górnik-Durose, 2016).

### Materialism, personality, and financial behavior

Materialism relates to different aspects of personal finance and money. More materialistic people tend to spend more money, have a higher amount of debt (Garðarsdóttir & Dittmar, 2012), and need more money to satisfy their needs (Richins & Dawson, 1992). Generalizing personal, cultural, and economic dispositions towards money, M. Tatzel proposed a ‘‘money worlds” theory based on two strategies of personal financial behavior - tight and loose – as the core of an individual’s economic behavior. These strategies, in combination with materialism level, describe four types of consumers. The Value-seeker tries to find the best low price and compare prices. The Non-spender worries about budget and is ready to sacrifice product quality. The Big Spender enjoys spending money on luxury and high-quality goods, and the Experiencer tends to spend for self-development and recreation. Each approach can be regarded as an adaptation strategy, but when extreme, these values may be dysfunctional (Tatzel, 2002). The differences between the “Peacocks” and “Mice” materialism-personality types in money spending were found for attitudes toward money, spending preferences, and the importance of brand (Górnik-Durose & Pilch, 2016).

In a series of studies, G. Donnelly and colleagues (2012) examined how money management, savings, debt, and compulsive buying are predicted by the Big Five traits and materialistic values. Across these studies, more materialistic people, especially when they believed that materialistic possession provides happiness, had poor money management. Among the Big Five traits, conscientiousness played the leading role in predicting money management (Donnelly et al., 2012). Among the Dark Triad traits, narcissism and psychopathy correlated with overall earnings (Jonason et al., 2018), risky money behavior (Crysel et al., 2013), and gambling (Jones, 2013), while Machiavellianism had a weak correlation or was uncorrelated with money- related features (gambling, risk) because of its commitment to strategy and long-term planning (Jones & Paulhus, 2009).

### Research problems in the present study

Following the “money worlds” theory by M. Tatzel (2002), the model of materialism proposed by Gornik-Durose and Pilch (2016), and the associations between materialism and the Dark Triad (Pilch & Górnik-Durose, 2016), the current study tried to investigate the potential combination of materialism values and Dark Triad traits. The combination of endorsement of materialistic values with Machiavellianism, narcissism, and psychopathy may not only clarify the features of materialistic individuals, but also the non-materialistic. Depending on the types of materialism-Dark Triad combinations, it can be possible to identify the different strategies in personal money management.

Therefore, we posed the following research hypotheses:

RQ 1. The same set of analyses used by Gornik-Durose and Pilch (2016) can be expected to distinguish groups of people with different combinations of materialism and the Dark Triad traits. Additionally, we assumed that the groups would demonstrate differences in Machiavellianism, narcissism, and psychopathy, as well as in facets of materialism that shed light on the group’s characteristics.

RQ2. Considering the correlations between Dark Triad traits and materialism facets, and the different incremental predictive validity of Dark Triad traits and materialism facets reported in prior research, it is possible to specify the different correlation patterns in potential subgroups.

RQ3. Given the links between various financial behaviors, materialism, and personality, is it fair to anticipate differences in personal financial control and financial responsibility across the potential subgroups?

These research questions were tested with cluster analysis and discriminant function analysis, correlation analysis, and ANOVA on the software R (R Development Core Team 2013)

## Methods

### Participants

The study sample included 442 undergraduate students from local universities age 18 to 25 (M = 20.7, SD = 1.67; 83% female). Participants received the link to an online survey consisting of self-report measures, demographic details, and questions assessing their Dark Triad traits, materialism, and money management. Each participant was informed of the nature of the study, and signed an online letter of informed consent. The study procedure complied with the ethical research code of the institution where the participants were recruited.

### Procedure

#### Questionnaires

##### The Dark Triad traits

The Dark Triad traits were assessed using the Russian version of Short Dark Triad measure (Egorova et al., 2015; Jones & Paulhus, 2014). The questionnaire consists of 27 items, nine for each of the Dark Triad traits: 1) Machiavellianism (*e.g*., “I like to use clever manipulation to get my way”); 2) narcissism (*e.g*., “I like to get acquainted with important people”); and 3) psychopathy (*e.g*., “People who mess with me always regret it”). Participants indicated their agreement with each statement using a five-point Likert type scale (1 = strongly disagree; 5 = strongly agree). The items were averaged to create indicators of narcissism, Machiavellianism, and psychopathy.

##### Materialism

The Russian version of the Material Values Scale (Khashchenko, 2016; Richins, 2004) was used to measure materialism and its dimensions. The scale consists of 12 items (4 items for each subscale). The answer was given on a 5-point scale (from 1 = strongly disagree to 5 = strongly agree).

The Success sub-scale assesses a person’s perception of possessions as markers of life success and achievement (*e.g*., “I admire people who own expensive homes, cars, and clothes”). The Centrality sub-scale assesses how important it is to pursue and acquire material goods as a primary objective in life (“I like a lot of luxury in my life”). The Happiness sub-scale assesses a person’s belief in the ability of material possessions to bring happiness (“I’d be happier if I could afford to buy more things”). To create indices of centrality, happiness, and success, the corresponding items were averaged. All items were averaged to create an index of materialism.

##### Money management

Participants completed the Money Management Scale (MMS; Donnelly et al., 2012) to assess their financial practices, and control and responsibility over their incomes and expenses. The scale measured participants’ sense of financial responsibility (*e.g.,* “When I reflect on my past buying behavior, I have been most likely to overspend” [reverse-coded]) and the degree to which they monitor their financial accounts (*e.g*., “Some people strive for financial clarity: knowing account balances, monthly expenses, loan interest rates, fees and fines”). Participants used seven-point Likert-type scales ranging from 1 (not at all) to 7 (a great deal). The average of all items and for each parcel (financial responsibility and financial control) were calculated.

## Results

### Materialism and the Dark Triad configuration

The initial exploratory phase of the analysis was to identify homogeneous groups of people contrasting with one another in terms of both the Dark Triad and materialism.

**Figure 1.**
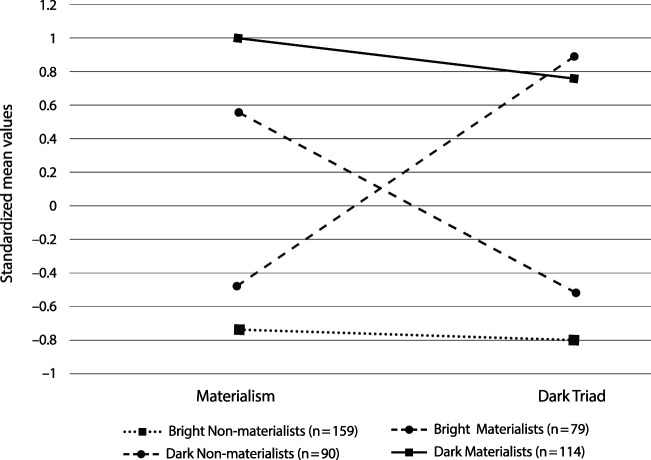
Materialism and Dark Triad configurations for the four groups

K-means cluster analysis (with Schwarz’s Bayesian Criterion) and two-step clustering were performed using standardized scores of the index of materialism scores along with the overall Dark Triad score (*Figure 1*). A four-cluster solution identified the following groups: 1) high scores in materialism and high overall Dark Triad; 2) low materialism and high overall Dark Triad; 3) high materialism but low overall Dark Triad; and 4) low scores in both materialism and overall Dark Triad.

The one-way ANOVA results indicated significant differences between clusters in relation to summed materialism scores (F (3, 438) = 182.6; p < .01) and overall Dark Triad score (F (3, 438) = 204.9; p < .01).

To simplify the description of groupings, we named the first group the Dark Materialists (N = 114); the second the Dark Non-materialists (N = 90); the third the Bright^1^ Materialists (N = 79); and the fourth the Bright Non-materialists (N = 159).

**Table 1:** Results of discriminant analysis

Discriminating variables	Wilks’ Lambda	F	β			Total structure coefficients		
			1	2	3	1	2	3
Machiavellianism	.61	93.37***	.529	–.240	.010	**.518**	–.309	–.049
Narcissism	.74	52.77***	.283	–.438	.505	.303	–.381	**.433**
Psychopathy	.67	73.67***	.363	–.363	.192	**.429**	–.357	.212
Success (materialism)	.60	97.49***	.448	.251	–.398	**.542**	.174	–.461
Happiness (materialism)	.73	53.04***	.303	.083	–.476	.376	.067	**–.615**
Centrality (materialism)	.55	118.43***	.400	.768	.483	.319	**.756**	.549

*Bold values indicate dominant variables in Function 1, Function 2, and Function 3.* Note: N = 442; *** —p < .001

Next, discriminant function analysis was performed to examine the underlying differences between the four groups identified in the previous analysis. The scores on the three MVS subscales (centrality, success, and happiness) and the three Dark Triad traits (Machiavellianism, narcissism, and psychopathy) were used to discriminate the groups (see the λ coefficients in *Table 1*). Function 1 had 64.1% of the variance (eigenvalue = 2.04; canonical correlation = .819; Wilk’s λ = .142, χ^2^ = 852.06, df = 18, p < .001). Function 2 contained 30% of the variance (eigenvalue = .955; canonical correlation = .70; Wilk’s λ = .431, χ^2^ = 367.26, df = 10, p < .001). Function 3 had only 5.9% of the total discriminating power (eigenvalue = .187; canonical correlation = .397, Wilk’s λ = .842, χ^2^ = 74.87, df = 4, p < .001).

The total structure coefficients showed that the material value of success, Machiavellianism, and psychopathy were dominant variables in Function 1. The material value of centrality was the dominant variable in Function 2. With Function 3, the main variables were the material value of happiness and narcissism (in opposition to each other).

Function 1 indicated a distinction between the materialistic value of acquisition as a primary predictor of success, and its correlations with manipulativeness and non-clinical psychopathy. Function 2 revealed a further disparity between high and low participants in the materialistic values of acquiring material possessions as a primary life goal (centrality). Function 3 indicated low happiness as a materialistic value of acquisition along with narcissism.

The analysis correctly classified an overall 91% of cases; 96.2% of cases low in materialism and low in Dark Triad (Bright Non-materialists); 86.1% of cases high in materialism and low in Dark Triad (Bright Materialists); 86.7% of cases low in materialism and high in Dark Triad (Dark Non-materialists); and 90.4% of cases high in both materialism and Dark Triad (Dark Materialists).

### Group differences in the materialism facets and the Dark Triad traits

Next, the one-way ANOVA and post hoc comparison were used to reveal the differences in materialism values scales (success, happiness, and centrality) and Dark Triad traits (Machiavellianism, narcissism, and psychopathy). The results indicated significant differences between the four groups in relation to the most listed traits (p < 0.05). The means and standard deviations are presented in *Table 2.*

**Table 2:** Descriptive statistics for study variables

Variables	Total sample (N=442)		Bright Non-materialists (N=159)		Bright Materialists (N=79)		Dark Non-materialists (N=90)		Dark Materialists (N=114)	
	*M*	*SD*	*M*	*SD*	*M*	*SD*	*M*	*SD*	*M*	*SD*
Machiavellianism	3.32	.54	2.97	.43	3.10	.48	3.59	.39	3.74	.39
Narcissism	3.06	.57	2.77	.51	2.87	.42	3.51	.38	3.24	.58
Psychopathy	3.00	.48	2.71	.38	2.82	.34	3.32	.38	3.28	.44
Success (materialism)	3.09	.73	2.64	.62	3.13	.57	2.93	.57	3.82	.50
Happiness (materialism)	3.45	.78	3.14	.73	3.31	.69	3.26	.67	4.12	.54
Centrality (materialism)	2.44	.92	1.93	.53	3.53	.64	1.97	.69	2.77	.88
Financial (money management) responsibility	3.88	1.15	4.26	.96	3.52	1.07	3.95	1.20	3.56	1.25
Financial (money management) control	5.22	1.12	5.37	1.02	4.77	1.12	5.48	.82	5.10	1.35
Money (composite) management	9.10	1.87	9.63	1.54	8.30	1.75	9.43	1.69	8.66	2.20
Materialism (composite)	2.99	.57	2.57	.40	3.32	.36	2.72	.35	3.57	.39
Dark Triad (composite)	3.13	.39	2.82	.27	2.93	.20	3.47	.20	3.42	.29

All groups differed on the success subscale (F (3, 438) = 97.49; p < .001) except for the post hoc comparison between the Bright Materialist and Dark Non-materialist groups. The happiness scale differed (F (3, 438) = 53.3; p < .001) among all groups, but the effect only appeared when comparing the group with high materialism and high overall Dark Triad (Dark Materialists) with the other three groups. The centrality value differed among all groups (F (3, 438) = 118.4; p < .001), but post hoc analysis didn’t reveal any differences between the Bright Non-materialist and Dark Non-materialist groups.

Machiavellianism (F (3, 438) = 97.37; p < .001) and narcissism (F (3, 438) = 52.77; p < .001) differed among all groups. The comparison of the psychopathy scores showed the differences (F (3, 438) = 73.67; p < .001), except for the post hoc comparison of the Bright Materialists and Non-materialists, with the Dark Materialists and Non-materialists.

### Correlation of the Materialism facets and the Dark Triad traits

The zero-order correlations between the materialism values scales and Machiavellianism, narcissism, and psychopathy were calculated for the groups extracted in the previous cluster analysis. The analysis was made for each group, and the same correlations of the difference between two independent correlation coefficients were tested using the technique described by Cohen et al., (2002). Narcissism positively correlated with material possession as a marker of success for groups of Bright Non-materialists (r = .17, p < .05), Bright Materialists (r = .23, p < .05), and Dark Materialists (r = .22, p < .05). There were no significant differences in correlations within each group (z = –.42:.07, p < .67:.95).

Psychopathy negatively correlated with material possession as a marker of success in the Bright Materialist (r = –.31, p < .05) and Dark Non-materialist (r = –.26, p < .05) groups, but positively with the Dark Materialist group (r = .20, p < .05). The correlation within the Dark Materialist group significantly differed from the other two groups (z = –3.28: -3.07, p < .01:.001). A positive correlation between the centrality of material values and psychopathy was found in the Dark Materialist group (r = .20, p < .05).

### Materialism, and the Dark Triad shapes the Money management

Significant differences between the groups with different configurations of materialism and the Dark Triad were found in the overall money management scores (F (3, 438) = 13.18, p < .001) and their components, *i.e*., financial responsibility (F (3, 438) = 12.16, p < .001), and financial control (F (3, 438) = 7.48, p < .001).

Post hoc comparisons indicated that Bright Non-materialists and Dark Non-materialists differed from both Bright and Dark Materialists in money management. The same results pertained to the post hoc analysis of financial responsibility. The post hoc analysis for financial control showed differences between Bright Materialists and Bright and Dark Non-materialists.

## Discussion

Our primary research goal was to discover how the characteristics of materialism and the Dark Triad combine to affect money management. The four groups were separated along the axes of materialism and negative personality characteristics (Machiavellianism, narcissism, and psychopathy). Despite the general differences in overall materialism and the Dark Triad, the combinations of features described different personality types.

The highest value of manipulativeness characterized the group of Dark Materialists. They evaluated their own and others’ success and happiness based on the number and quality of possessions acquired. Among the Dark Triad traits, their Machiavellianism was higher than their narcissism and psychopathy.

The Dark Non-materialists tended to exploit people and manipulate others for personal benefit, and they had a stronger sense of entitlement and superiority to others than the Dark Materialists. On the other hand, material things did not have a key position in their lives and did not serve as their primary source of happiness or discontent.

In manipulativeness, feeling of superiority, and cold-bloodedness, Bright Materialists and Non-materialists exhibited the same low values, but a difference arose in their materialistic values. In contrast to the Bright Materialists, the Bright Non-materialists did not put material possession at the center of their lives, nor did they judge their level of success by the amount and quality of goods they had collected.

Concerning the associations between Dark Triad traits and facets of materialism, the association of narcissism with material possession as a marker of success characterized the two materialistic groups, as well as the Dark Triad level and the Bright Non-materialist group. This finding lends credence to the idea that narcissism may be the root of materialistic ownership, as previously proposed (Górnik-Durose & Pilch, 2016). For comparison, for groups of Bright Materialists and Dark Non-materialists, more self-control, reduced impulsiveness, and boldness were correlated with a lower assessment of their success in gaining tangible possessions. In contrast, the prevalence of psychopathic characteristics in Dark Materialists resulted in a higher evaluation of success in terms of financial gain and a greater emphasis on material possessions.

Thus, narcissism may be viewed as a necessary personality feature for those with solid materialistic priorities. The combination of solid narcissism and psychopathy increases attachment to material possessions, indicating their value as signals for the Dark Triad individuals in gaining status and domination. An evolutionary viewpoint may be used to investigate the relationship between materialism and the Dark Triad (Pilch, & Górnik-Durose, 2016). The link between persons possessing strong Dark Triad features and their ability to accumulate greater wealth could be viewed as a sign of qualities that may be inherited by their offspring. Consequently, those qualities become attractive in the eyes of a potential partner and deter rivals from competing.

Comparing money management in groups with various combinations of the Dark Triad traits and materialism leads to the assumption that low materialistic values are preferable for accounting and comprehending one’s current financial situation, purchase planning, and saving. In detail, the combination of a robust materialistic drive and a low level of negative personality traits may lead to financial disorganization. Individuals with a high materialistic orientation and high Dark Triad traits demonstrated low financial responsibility but reasonable financial control. The most financially responsible and controlling were non-materialistic people with low Machiavellianism, narcissism, and psychopathy, followed by the individuals with the combination of low materialistic orientation and high Dark Triad traits.

We can also analyze our results in the context of life history theory (Figueredo et al., 2005). Research within that theoretical framework has shown that Dark Triad traits are positively associated with the fast spectrum of life history strategies (Pilch, & Górnik-Durose, 2016). Individuals with high levels of Dark Triad traits use materialism as a strategic tool to make quick life trade-offs, which explains the poor money management in groups with high materialism and high Dark Triad traits. The pursuit of material possessions, combined with an antagonistic personality, led to acquiring and owning goods with a short-term outlook, lacking long-term planning. On the other hand, the lower the level of materialism (even if the Dark Triad traits are high), the better the financial management as a part of the slow life history strategy.

A direct comparison of our results with the materialism-personality types discovered by Górnik-Durose and Pilch (2016) and the “money worlds” types identified by M. Tatzel (Tatzel, 2002) would be speculative, but based on key characteristics of the types, we may propose the following. Overall, materialists are receptive to externals (appearances and status) and self-centered (less giving, less concerned with relationships than those lower in materialism), qualities which also characterize individuals with high Dark Triad traits. According to our results, people with high Machiavellianism, narcissism, and psychopathy may have opposite materialistic values. Dark Materialists may be compared to “Peacocks,” whereas Bright Materialists can be compared to “Mice.” Even though Dark Non-materialists have the highest level of narcissism they couldn’t be compared directly with “Peacocks” type because of low materialistic values.

Taking into account the distinctions in money management across the different Dark Triad — materialism groups, the other possible comparison is with M. Tatzel’s consumer styles. Bright Non-materialists are closer to Non-Spenders because they are in control of their budget and spending, just as they are in control of themselves, and are not motivated by the need to influence or impress others. While Dark Non-materialists are good at money management, they tend to manipulate people and show off their superiority. Such a personality type is comparable to the Experiencer in terms of seeking status, power, and prestige through outward excessive spending but not through tangible possessions. Although Dark Materialists are similar to the Value seeker in terms of acquisition as the pursuit of happiness and possession-defined success, they are not particularly adept at money management. However, their tendencies to manipulate others and show off , as well as their lack of control, place them close to the Experiencer type. Due to their strong materialism beliefs and poor money management, particularly in financial control, Bright Materialists might be likened to the Big Spender type.

## Conclusion

This study aimed to determine the relationship between materialistic ideals and unpleasant personality characteristics. When materialism and personality characteristics were combined, we gained more evidence of the disparities in personal money management practices. Machiavellianism and narcissism were more prevalent in the Bright and Dark Materialists than in the Non-materialists, and materialistic ideals were more strongly endorsed in the Dark Materialists. Regarding the “dark” personalities, it is possible to argue that materialism may be viewed as a strategy to obtain resources, position, and prestige at the expense of others, as shown by the specific pattern of correlation between them. Interestingly, the combination of high materialism and low Dark Triad characteristics resulted in the weakest financial control. Contrary to this, the combination of low materialism and high Dark Triad qualities resulted in better money management, which gives credence to the concept that the Dark Triad traits might improve financial behavior.

## Limitations

To begin with, the research was cross-sectional. However, due to the exploratory nature of the study, the collected data may serve as the initial body of evidence for future studies. Second, the use of self-report methods in accounting was limited by the cross-sectional design. Self-report measures are commonly used in studies focusing on personality traits and materialism. Third, the sample consisted of more than 80% females, thus limiting its generalizability. In addition, there was an unequal distribution of sexes among the subgroups. The group with high materialism and low Dark Triad traits contained the smallest percentage of men (5%), while men made up 29% of the other three groups. Future studies might attempt to avoid this issue by forming a more balanced sample and using objective measures of financial behavior.

## Footnotes

1 We use the word “bright” to identify the groups with low values of Dark Triad traits; it is not to be confused with the Light Triad of personality proposed by S.B. Kaufman (Kaufman, S.B., Yaden, D.B., Hyde, E., & Tsukayama, E. (2019). The Light vs. Dark Triad of Personality: Contrasting Two Very Different Profiles of Human Nature. Frontiers in Psychology, 10, 467. https://doi.org/10.3389/fpsyg.2019.00467)
